# Stimulating or constraining creativity? Traditional vs. generative AI on divergent thinking in product design

**DOI:** 10.3389/fpsyg.2026.1839565

**Published:** 2026-06-30

**Authors:** Huan Lin, Letian Xie

**Affiliations:** 1 College of Mechanical Engineering, Quzhou University, Quzhou, China; 2 College of Mechanical Engineering, Zhejiang University of Technology, Hangzhou, China

**Keywords:** creativity, divergent thinking, generative AI, ideation methods, product design

## Abstract

**Introduction:**

The rise of generative AI (GenAI) has brought both opportunities and challenges to product design, raising the question of whether it stimulates or constrains creativity. This study empirically examines the effects of traditional and GenAI-assisted ideation methods on cognitive creativity and perceived creativity support during the divergent thinking stage of product design.

**Methods:**

Focusing on both the creativity of design outcomes and designers' self-reported user experience, a mixed-methods experiment was conducted with 41 sophomore students majoring in product design. Three ideation methods were compared: unaided, Internet-assisted, and GenAI-assisted.

**Results:**

The results revealed that, among novice product design students, traditional methods more effectively enhanced divergent thinking performance, whereas GenAI-assisted methods significantly improved designers' perceived creative support, specifically on usability, exploration, and immersion during the ideation process. Moreover, no significant differences were observed among participants with varying levels of creative tendency.

**Discussion:**

These findings suggest that, although GenAI has not yet surpassed traditional approaches in generating highly original design outcomes, it serves an important facilitative role in design education by enriching learners' creative perception and affective engagement. The study provides empirical evidence for the thoughtful integration of GenAI into the design process and highlights the need for future research on optimizing human-AI collaboration to augment, rather than replace, human creativity in design learning and practice.

## 1 Introduction

Creativity is broadly recognized as a cornerstone of innovation and a key driver of progress across industries (Caliendo and Rodríguez, 2024; Cropley, 2006). In product design, creativity is especially vital during the early ideation stage, when designers seek to generate a broad range of novel and valuable concepts that can differentiate products in competitive markets (Childs et al., 2022). Enhancing designers' divergent thinking (Guilford, 1967), the ability to explore multiple ideas and directions, is central to fostering innovative product solutions and increasing the likelihood of market success (Xie, 2023). Consequently, understanding how different ideation methods support and stimulate divergent thinking has become a key concern for both researchers and practitioners, as it determines how effectively designers can translate ideas into tangible outcomes.

Over the years, various ideation methods have been developed to enhance the design process, with their differences becoming particularly evident in the early stages of design (Gero et al., 2012). Traditional approaches such as brainstorming (Wilson, 2013), internet searching (Oliva and Storm, 2023), and mind mapping (Crowe and Sheppard, 2012; Cheng and Zhang, 2025) have long served as core techniques in early-stage design practice. More recently, the rapid advancement of artificial intelligence, especially large language models (LLMs) and generative AI (GenAI), has brought transformative opportunities to design ideation (Cao et al., 2023). These technologies utilize machine learning and pattern recognition to generate visual concepts and problem-solving frameworks from designers' prompts, enabling rapid exploration of alternative directions and expanding the scope of creative inquiry.

However, existing evidence regarding the impact of GenAI on design creativity remains mixed. Some studies suggest that GenAI can support divergent thinking by broadening conceptual exploration, improving design quality, and reducing cognitive load during ideation (Chandrasekera et al., 2025; Chiou et al., 2023; Li and Li, 2024). These studies suggest that GenAI can function as a form of creative support that enhances ideation efficiency and exploratory thinking. In contrast, other studies have raised concerns that GenAI may increase design fixation and reduce originality, particularly in complex design tasks (Cheng and Zhang, 2025; Wadinambiarachchi et al., 2024). Taken together, these findings indicate that the influence of GenAI on creativity is not uniformly positive or negative, but may depend on the specific ideation context.

Despite growing interest in GenAI-assisted ideation, important gaps remain in the current literature. First, relatively few studies have systematically compared traditional and GenAI-assisted ideation methods within controlled divergent thinking tasks, particularly in the context of product design education. Second, prior research has primarily focused on design outcomes related to cognitive creativity, while designers' subjective experiences during ideation have received much less attention. In addition, the role of individual differences in shaping responses to different ideation methods remains insufficiently understood. Given the limited research examining how GenAI influences creativity in educational settings (Benvenuti et al., 2023), the present study addresses these gaps by investigating how traditional and GenAI-assisted ideation methods influence both creative performance and perceived creativity support during divergent thinking in product design.

**Figure 1:**
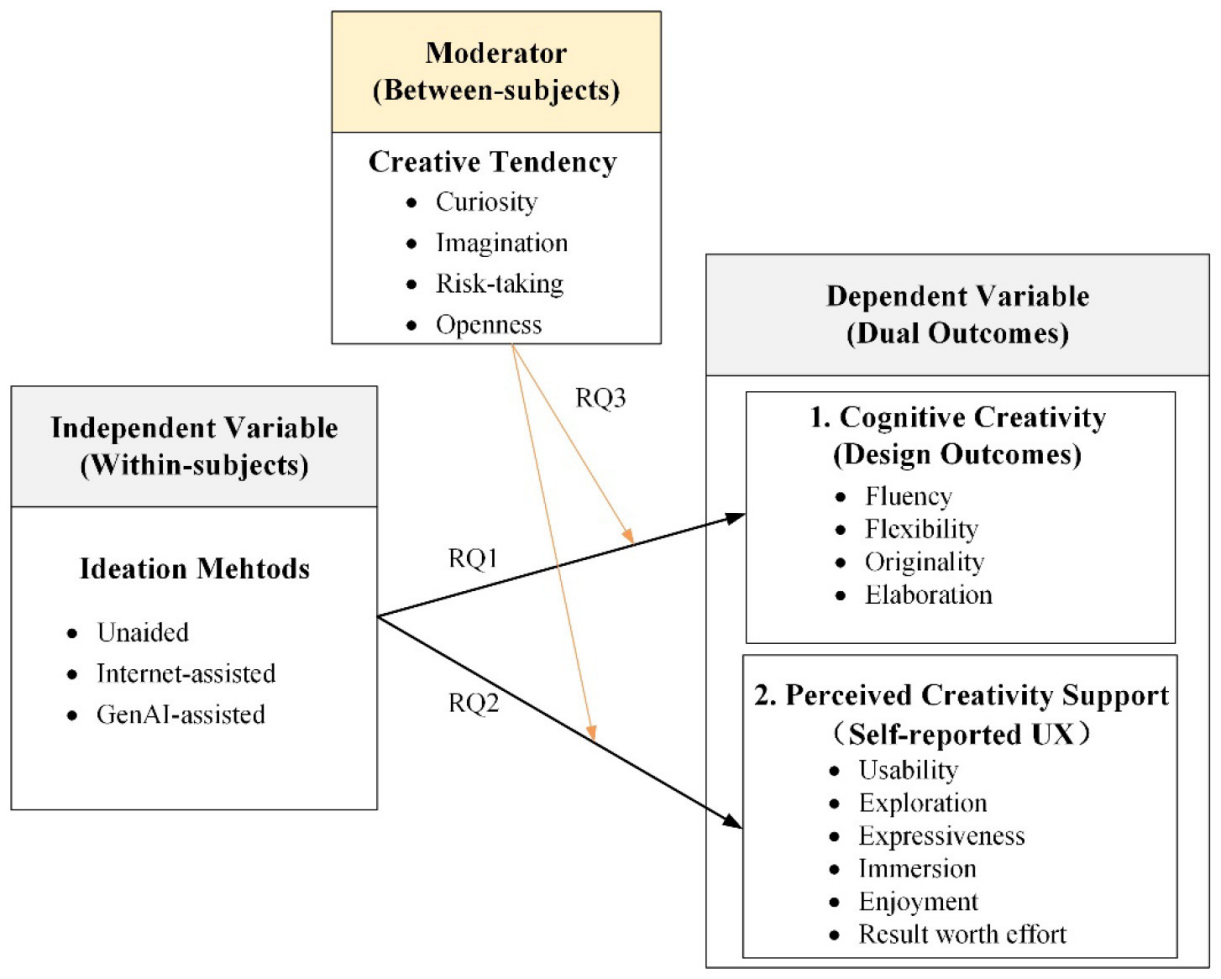
Dual-path conceptual framework distinguishing cognitive creativity from perceived creativity support during divergent thinking.

As illustrated in Figure 1, the present study adopts a dual-path framework consisting of cognitive creativity and perceived creativity support. Cognitive creativity is reflected in design outcomes and assessed through Guilford (1967) four dimensions of creativity, namely fluency, flexibility, originality, and elaboration, using the Consensual Assessment Technique (CAT; Amabile, 1982). Perceived creativity support refers to designers' subjective evaluations of the extent to which an ideation method facilitates the creative process and is measured using dimensions adapted from the Creative Support Index (CSI; Carroll et al., 2009; Cherry and Latulipe, 2014). Creative tendency is further incorporated as a potential moderating variable to examine whether individuals with different creative dispositions respond differently to traditional and GenAI-assisted ideation methods.

The proposed framework enables a comprehensive evaluation of both objective design performance and subjective creative experience, thereby addressing key gaps identified in previous research.

To guide the investigation, the following research questions are proposed:

- RQ1: How do traditional and GenAI-assisted ideation methods affect the creativity of design outcomes during divergent thinking?
- RQ2: How do these ideation methods influence designers' perceived creativity support?
- RQ3: Do designers with different levels of creative tendency respond differently to traditional and GenAI-assisted ideation methods?

This study contributes to a deeper understanding of how traditional and GenAI-assisted ideation methods influence creativity in the design process. By comparing unaided, internet-assisted, and GenAI-assisted approaches, the study investigates their effects on both the creativity of design outcomes and designers' perceived creativity support. The findings are expected to provide theoretical insights into the role of GenAI in divergent thinking and to offer practical implications for the effective integration of AI tools into product design education and professional design practice.

## 2 Literature review

### 2.1 Creativity and divergent thinking

Creativity is commonly defined as the ability to produce outcomes that are both novel and appropriate. It encompasses both the process of generating ideas or solving problems and the outcomes that emerge as products or solutions (Amabile, 1983; Amabile et al., 2005; Sternberg, 1988; Weisberg, 1988). In product design, creativity serves as a fundamental driver of original concept development and innovation (Howard et al., 2010). It involves both cognitive and affective components, which collectively contribute to the emergence of creative thought and behavior (Chen et al., 2020; Williams, 1993).

Divergent thinking, a key aspect of cognitive creativity, is described as the ability to produce diverse, novel solutions in response to open-ended problems (Guilford, 1967; Tan and Luhrs, 2024; Wu and Chen, 2019). It involves generative and associative reasoning processes (Lazar, 2018) and is widely recognized as an indicator of creative potential (Runco and Acar, 2012). Within the domain of product design, divergent thinking is particularly critical during the early stages of ideation and concept development, as the breadth and originality of ideas generated at this stage can substantially influence subsequent innovation outcomes (Li et al., 2023).

Taken together, the literature on divergent thinking in design highlights two major insights. First, divergent thinking is consistently operationalized through the dimensions of fluency, flexibility, originality, and elaboration (Guilford, 1967), providing a robust and widely accepted measurement framework across studies. Second, external stimuli, including analogical reasoning, bio-inspired tools, and digital references, can effectively support divergent thinking processes (Chen et al., 2024). However, their effectiveness is closely related to task characteristics and the timing of their introduction during the ideation process (Lee, 2019).

Despite these advances, previous research has primarily emphasized cognitive outcomes, while devoting comparatively less attention to designers' subjective experiences throughout the divergent thinking process. Consequently, the extent to which designers perceive different ideation methods as supportive of creativity remains insufficiently explored. This gap motivates the present study to incorporate perceived creativity support as a complementary outcome dimension alongside cognitive creativity, thereby enabling a more comprehensive understanding of creativity in design ideation.

### 2.2 Ideation methods: traditional vs. generative AI

With digital technological advancements, ideation methods in product design can generally be categorized into traditional and GenAI-assisted approaches.

Traditional ideation methods have been widely applied and studied in design research, although their effectiveness often depends on the specific context in which they are used. Brainstorming, long regarded as a fundamental ideation technique (Wilson, 2013), can effectively stimulate idea generation when appropriate creative prompts or stimuli are introduced (Howard et al., 2010). However, its effectiveness may decline when participants become overly focused on their initial ideas, thereby limiting further exploration and reducing the diversity of potential solutions.

Internet searching presents a similar dual effect. On the one hand, it provides designers with access to extensive information and inspiration, which can broaden the scope of idea generation (Oliva and Storm, 2023). On the other hand, exposure to highly similar online content may guide participants toward commonly encountered ideas, reducing originality and increasing similarity among design outcomes (Oliva and Storm, 2023; Oppenheimer and Patterson, 2025). These findings suggest that the effectiveness of traditional ideation methods depends not only on the method itself, but also on how it is implemented and the cognitive demands associated with the design task. This issue becomes particularly important when traditional approaches are compared with emerging GenAI-assisted ideation methods.

GenAI tools, particularly text-to-image and multimodal systems such as DALL·E, Midjourney, and Stable Diffusion, have recently transformed creative workflows (Azzola et al., 2025; Yin Y. et al., 2023). Unlike traditional methods that depend solely on human ideation, GenAI can instantly produce diverse visual and conceptual outputs from textual prompts, thereby facilitating exploration beyond the boundaries of individual imagination (Chandrasekera et al., 2025; Elgammal et al., 2017; Wang et al., 2025).

Existing studies report mixed findings regarding the impact of GenAI on creativity. Tan and Luhrs (2024) utilized Midjourney, a text-to-image GenAI tool, to support architectural design. Their findings showed that Midjourney can significantly enhance creative thinking, particularly by enabling the rapid generation and refinement of design concepts. Jeon et al. (2021) introduced the GenAI tool FashionQ to assist in fashion design and demonstrated its effectiveness in promoting both divergent and convergent thinking. In contrast, Wadinambiarachchi et al. (2024) reported that GenAI support during ideation could increase design fixation on initial examples, leading participants to generate fewer ideas that were less varied and lower in originality compared with a baseline condition without inspiration support.

Taken together, previous studies suggest that the influence of GenAI on design creativity involves both opportunities and challenges. While some research emphasizes its ability to stimulate novel ideas and expand creative exploration, other studies point to the risk of constraining originality and increasing design fixation (Doshi and Hauser, 2024). The impact of GenAI therefore appears to depend on multiple factors, including the nature of the design task (Simkute et al., 2025; Wadinambiarachchi et al., 2024), the specific type of tool being used (Wang et al., 2025), and the ways in which it is integrated into the design process (Yu, 2025). During early-stage design, traditional and GenAI-assisted methods each offer distinct strengths for supporting divergent thinking. A systematic understanding of their comparative effects, as well as designers' experiences with these approaches, is essential for optimizing creative processes in product design education and professional practice.

### 2.3 Creative tendency

Creative tendency refers to an individual's inherent inclination to engage in creative thinking and behavior, encompassing personal traits such as curiosity, imagination, risk-taking, preference for complexity, openness to experience, extraversion, and related characteristics (Chen et al., 2020; Rassouli-Baghi, 2024). While prior work has sometimes classified these traits under affective creativity (Chen et al., 2020), the present study conceptualizes creative tendency as an individual difference variable that is distinct from both cognitive creative ability and situational affective states. This distinction aligns with Williams (1993) and Feist (1999), who positioned creative tendency as a motivational disposition that influences engagement across contexts, rather than as a component of momentary emotional experience.

A relatively consistent finding in personality and creativity research is that traits such as openness to experience, curiosity, and risk taking are positively associated with creative performance (Feist, 1998; Rassouli-Baghi, 2024; Silvia et al., 2009). This relationship has been observed across different domains and age groups, suggesting that creative tendency functions as a relatively stable motivational disposition (Eysenck, 1993; Williams, 1993).

However, recent studies have introduced an important nuance to this relationship. Emerging evidence suggests that the influence of creative tendency may become weaker when external tools provide substantial creative support during the ideation process. For example, Doshi and Hauser (2024) found that access to GenAI tools benefited less creative writers more strongly than highly creative writers, thereby reducing the performance gap between the two groups. This compensatory effect indicates that creative tendency may not influence creative performance uniformly across different ideation conditions, although this possibility has rarely been examined directly in previous research.

To date, few studies have systematically examined how individuals with different levels of creative tendency perform across traditional ideation methods, such as unaided thinking and internet searching, and GenAI-assisted ideation methods in product design contexts. It therefore remains unclear whether creative tendency amplifies the benefits derived from GenAI tools or whether GenAI can compensate for lower levels of creative tendency. To address this gap, the present study treats creative tendency as an exploratory moderating variable for examining its role across different ideation conditions.

## 3 Methods

This study empirically investigates the effects of traditional and GenAI-assisted ideation methods on divergent thinking in product design. To provide a comprehensive assessment of creativity, both cognitive creativity and perceived creativity support were examined, focusing on the creative quality of design outcomes and designers' subjective evaluations of creativity support. In addition, the moderating role of creative tendency was explored to determine whether individuals with different creative dispositions respond differently to these ideation methods.

### 3.1 Participants

Prior to data collection, an *a priori* power analysis conducted with G^*^Power (Faul et al., 2009) revealed that a minimum of 36 participants was required to achieve a statistical power of 0.80, assuming a medium effect size (*f* = 0.25) and an alpha level of 0.05.

Given the recent emergence of GenAI tools in design education, participants' prior experience with GenAI-assisted ideation was assessed *via* a pre-experiment questionnaire. Results indicated limited familiarity with GenAI tools in product design contexts: 25 participants (60.9%) reported never having used GenAI for design ideation, 10 (24.4%) had used such tools 1–2 times out of curiosity, 5 (12.2%) used them occasionally (monthly) for coursework, and only 1 participant (2.4%) reported weekly usage. Mean self-rated familiarity with text-to-image GenAI on a 7-point scale was 2.08 (SD = 0.92), and mean prompt-writing confidence on a 7-point scale was 2.12 (SD = 1.15), indicating generally low proficiency. No participant had received formal training in prompt engineering prior to the experiment, and none reported using Doubao specifically for product design ideation.

To assess participants' creative tendency, all students enrolled in a Product Creative Design course were invited to complete the Creativity Assessment Packet (Williams, 1993), a 50-item self-assessment that measures four subdimensions: curiosity, imagination, risk-taking, and openness to new experiences. Scores were interpreted according to established criteria: scores above 135 indicate excellent creativity, scores between 120 and 134 indicate good creativity, scores between 90 and 119 indicate average creativity, and scores below 90 indicate low creativity. Participants completed the assessment by self-rating their responses based on their personal perceptions and experiences.

The results showed that 20 participants scored within the good creativity range (120–134), and 21 participants scored within the average creativity range (90–119). No participants fell into the excellent or poor creativity categories. Given the relatively homogeneous educational background of the sample, additional participants from other creativity categories were not recruited. Consequently, only the average and good creativity groups were included as levels of the between-subjects factor.

A total of 41 second-year undergraduate students majoring in product design participated in the experiment (mean age = 19.5 years, SD = 1.27). The studies involving human participants were reviewed and approved by Quzhou University's ethics committee in accordance with the principles of the American Psychological Association (APA). The studies were conducted in accordance with the local legislation and institutional requirements. The participants were not informed of the specific purpose of the experiment in advance and provided their written informed consent to participate in this study.

### 3.2 Design

The experiment employed a 3 × 2 mixed factorial design. The within-subjects independent variable was ideation method, with three levels: unaided (control), Internet-assisted (Baidu Images), and GenAI-assisted (Doubao). Baidu Images was selected as a representative image-search platform, whereas Doubao was selected as a representative multimodal GenAI system capable of generating textual and visual design inspiration from user prompts. The between-subjects factor was creative tendency level (average/good). The dependent variables were the creativity scores of the design outcomes and the perceived creativity support reported by participants under each ideation method (see Section 3.4 for operational definitions).

All experimental sessions were conducted individually in a laboratory environment. Materials included a computer, blank A4 paper, and pens. During the assisted conditions, participants were provided access to digital tools, including a pre-installed Baidu Images browser interface and the Doubao application.

To reduce potential confounding effects associated with differences in digital tool proficiency, several procedural controls were implemented. For the GenAI-assisted condition, standardized prompt-writing guidelines and a brief training session were provided to establish a comparable baseline level of prompt-writing competence, given participants' generally low self-reported proficiency (*M* = 2.12, SD = 1.15 on a 7-point scale). For the Internet-assisted condition, the Baidu Images interface was initialized with a blank search page, and participants were instructed to conduct keyword-based searches without using AI-generated recommendations or related search suggestions. These controls were designed to ensure that observed differences could be attributed primarily to the ideation methods rather than to variations in participants' familiarity with digital tools.

Participants completed a chair design ideation task, with the goal of generating as many ideas as possible within a fixed timeframe. The chair was selected as the design subject for two reasons. First, its familiarity in daily life makes it accessible to novice designers, facilitating brainstorming during divergent thinking. Second, it provides sufficient complexity to support idea generation while remaining manageable within the constraints of a timed task. For the digital-assisted conditions, standardized prompt guidelines were used to ensure procedural consistency.

Three product design experts, each with more than 10 years of professional or academic experience, assessed the creative outputs generated in the experiment and served as independent evaluators. Prior to the assessment, all design artifacts were anonymized and assigned identification codes by a research assistant to ensure objectivity. The evaluators were blind to the three experimental conditions (unaided, Internet-assisted, and GenAI-assisted ideation) as well as to the production phase of each design. All evaluations were conducted independently, without communication or consultation among the evaluators, using a standardized scoring rubric (see Section 3.4, Measures).

### 3.3 Procedure

**Figure 2:**
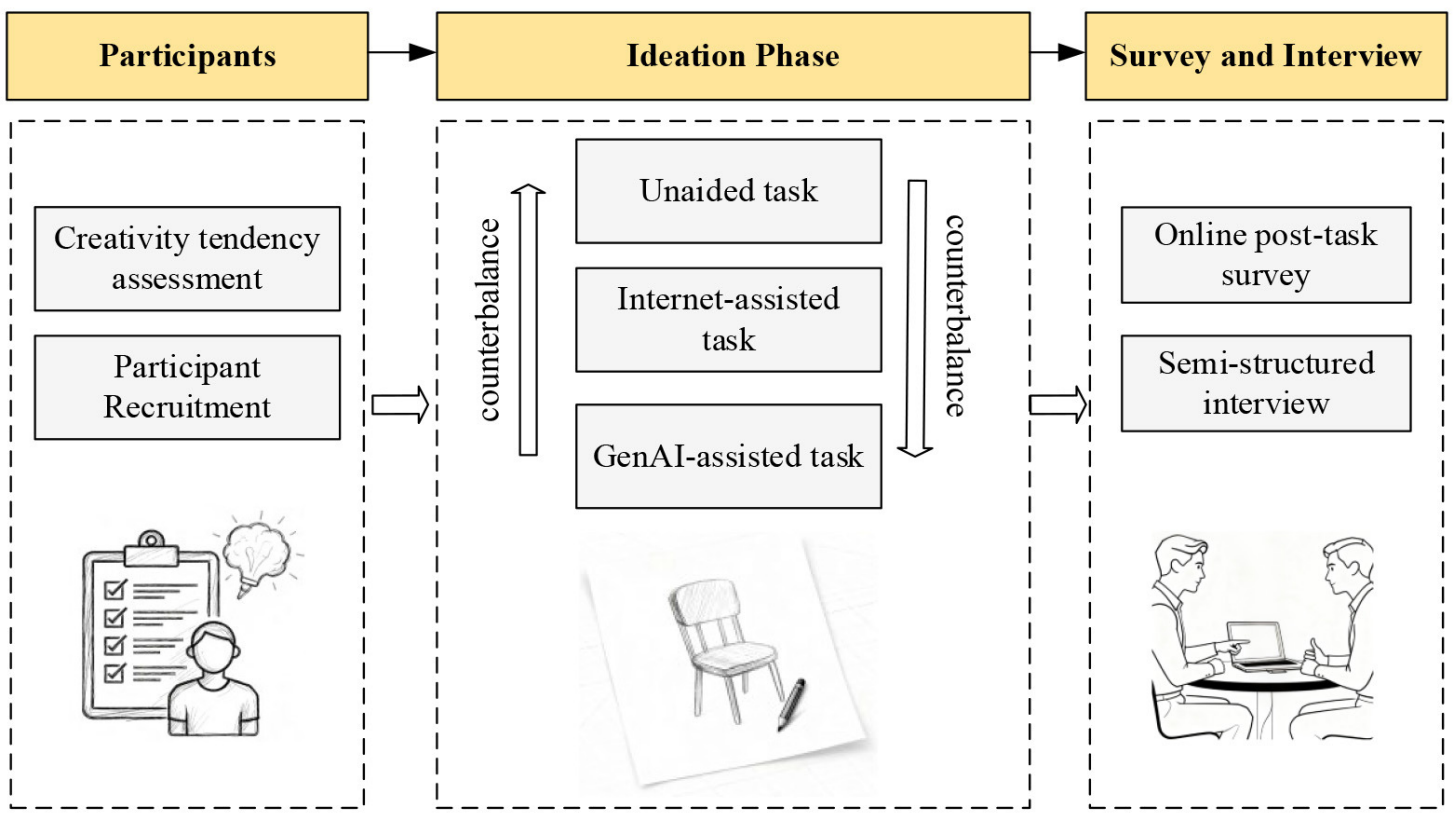
The overall experimental procedure.

Upon arrival at the laboratory, participants were welcomed and guided to individual workstations equipped with Dell Precision 5,510 computers. The session began with the completion of a demographic questionnaire, which collected information on gender, age, and prior experience with digitally assisted creative tools in product design ideation. Following a brief orientation, participants completed a sequence of three ideation tasks, each designed to evaluate the influence of different creative support methods (see Figure 2).

The first task, Unaided Ideation, consisted of a 10-min session during which participants generated chair design concepts using only freehand drawing. No external resources, such as mobile phones, computers, or reference materials, were permitted during this stage. The 10-min duration was informed by prior research (Beaty and Silvia, 2012; Jang et al., 2019; Toh and Miller, 2016), which indicates that creative ideation typically plateaus after approximately 9–10 min.

The second task, Internet-assisted Ideation, allowed participants to explore chair design ideas using designated online image searches as visual references. They were instructed to continue brainstorming and sketching for 10 min, utilizing online visual resources to support inspiration and concept development.

The third task, GenAI-assisted Ideation, involved the use of a designated text-to-image GenAI tool, specifically the Doubao. To control for individual differences in prompt engineering skill and AI familiarity, a standardized 5-min prompt tutorial was administered immediately before this condition. The tutorial covered: (1) basic prompt structure (subject description, style attributes, and functional requirements), (2) three chair-design examples (e.g., a minimalist wooden chair with curved backrest, Scandinavian style, ergonomic), and (3) one practice trial with corrective feedback. Given the uniformly low levels of prior GenAI experience reported by participants, the tutorial and standardized prompt materials were intended to establish a comparable baseline level of tool familiarity before the ideation task. During the task, participants were provided with a printed prompt reference card to reduce working-memory demands, and the Doubao interface was preconfigured with default generation parameters, with advanced settings disabled to ensure procedural consistency. A research assistant was present solely to address technical issues and did not provide any design-related or creative guidance. Within the 10-min ideation period, participants generated initial chair concepts using AI-generated images based on text prompts and subsequently refined or extended these concepts through freehand sketching.

To control for potential learning effects or order-related biases, the sequence of the three design tasks was counterbalanced across participants using a systematic rotation schedule. This approach helped ensure that any observed differences in creative performance could be attributed primarily to the ideation method rather than task order.

Throughout all tasks, participants were instructed to generate original chair design concepts specific to the assigned method. For each distinct concept, they were encouraged to include a written description detailing the source of inspiration, defining features, and any underlying design insights. Participants were encouraged to sketch as many concepts as possible and to clearly number each one to facilitate the quantification and analysis of idea fluency.

Upon completion of all ideation tasks, participants completed an online post-task survey designed to capture their subjective experiences with each ideation method. Finally, each participant engaged in a brief (approximately 5-min) semi-structured interview with the researcher. This session provided an opportunity for participants to reflect on and elaborate upon their experiences, offering qualitative insights into the cognitive and affective influences of the different ideation methods employed in the experiment.

### 3.4 Measures

To gain deeper insights into the interaction between users and ideation methods during the divergent thinking process, we focused on two key aspects: the creativity scores of the design outcomes and participants' perceived level of creativity support associated with each method (see Table 1).

**Table 1:** Measures of creativity of design outcomes and perceived creative support.

Creativity assessment	Methods	Measures	Detailed description of measures
Objects: design outcomes	CAT (consensual assessment technique)	Fluency	The number of distinct design ideas generated
		Flexibility	The variety of idea categories or conceptual shifts within the design.
		Originality	The uniqueness or novelty of the design solution compared to others.
		Elaboration	The level of detail, completeness, and refinement in the design output.
Subjects: perceived creative support	Self-report (creative support index)	Usability	The extent to which the ideation method is intuitive and easy to use.
		Exploration	The degree to which the method encourages users to pursue diverse or novel ideas.
		Expressiveness	The method's ability to help users clearly articulate and externalize their ideas.
		Immersion	The sense of deep engagement and focus during the ideation process.
		Enjoyment	The level of enjoyment or satisfaction experienced during the activity.
		Result worth effort	The perceived value of the creative outcome relative to the effort invested.

To evaluate the creativity of design outcomes, we adopted the Consensual Assessment Technique (CAT). CAT provides a straightforward yet robust approach for assessing product-based creativity (Amabile, 1982; Baer et al., 2004; Kaufman et al., 2008). Rather than relying on a predetermined theoretical definition of creativity, CAT addresses the inherently subjective nature of creativity by asking domain experts to evaluate the creative quality of a product. Because this technique closely mirrors real-world evaluations of creativity, it is widely regarded as the gold standard for creativity assessment and has been extensively used in prior product design studies (Freeman et al., 2015; Hu et al., 2023).

Given the lack of standardized indicators for assessing creativity, particularly in the early stages of divergent thinking (Laing and Masoodian, 2016), we adopted Guilford's (Guilford, 1967) four classic dimensions of divergent thinking: fluency, flexibility, originality, and elaboration. These metrics were adapted to evaluate participants' hand-drawn chair design concepts.

Fluency, defined as the total number of distinct ideas generated, measures ideation productivity by reflecting both the number of design sketches produced within the given time and the participant's active cognitive engagement (Basadur and Thompson, 1986; Shah et al., 2003; Verhaegen et al., 2013). Flexibility reflects the diversity of concepts across different categories or functional approaches, indicating the range of variation in both form and function. Originality evaluates the novelty and uniqueness of design concepts (Luo and Dong, 2017; Sternberg and Lubart, 1999), excluding inappropriate or unconventional ideas (Runco and Charles, 1993), and indicates how far participants' proposals diverge from existing solution (Laing and Masoodian, 2016). Elaboration captures the extent of detail and development in sketches, encompassing the refinement of individual design elements, the thoroughness of annotations, and the clarity of visual communication. It is commonly used to evaluate the richness and completeness of design outputs (Mahmoud et al., 2023; Said-Metwaly et al., 2018).

Fluency was assessed by counting the number of distinct design concepts provided by participants. The remaining criteria, flexibility, originality, and elaboration, were systematically assessed *via* a 5-point Likert scale, wherein 1 signified “poor,” 3 represented “average,” and 5 denoted “excellent,” in accordance with established methodologies (Demirkan and Afacan, 2012; Dong and Zhu, 2023; Goldschmidt and Smolkov, 2006).

The evaluation of participants' design outcomes was conducted by a panel comprising three experts: two with extensive professional experience exceeding 10 years in product design within a Chinese furniture enterprise, and one with over a decade of academic experience in design education at the university level. All experts served as independent evaluators and were blind to the three experimental conditions.

To assess participants' perceived creativity support across the three ideation methods, an online post-task questionnaire was administered. It is important to note that this construct captures participants' subjective perceptions of how effectively an ideation method facilitates their creative process. Conceptually, it is more closely related to user experience (UX) and creative self-efficacy than to creative ability or the objective quality of creative outcomes (Carroll et al., 2009; Cherry and Latulipe, 2014). The measurement framework was informed by the Creative Support Index (CSI), which has been widely used to assess users' subjective experiences with creativity support tools (Carroll et al., 2009; Cherry and Latulipe, 2014; Dewit et al., 2020). Drawing from prior research and tailored to the context of individual design ideation, we selected six dimensions for evaluation: usability, defined as the extent to which the ideation method is intuitive and easy to use; exploration, referring to the degree to which the method encourages users to pursue diverse or novel ideas; expressiveness, reflecting the method's ability to help users clearly articulate and externalize their ideas; immersion, capturing the sense of deep engagement and focus during the ideation process; enjoyment, representing the level of satisfaction experienced during the activity; and result worth effort, indicating the perceived value of the creative outcome relative to the effort invested. These dimensions collectively reflect how effectively each ideation method supports the creative process from the designer's perspective and are consistent with UX research on creativity support tools (Shneiderman, 2007). One CSI dimension, collaboration, was excluded because the present study focused exclusively on individual rather than collaborative ideation activities.

Participants assessed all six dimensions on a 7-point Likert scale, where 1 meant “not at all,” 7 meant “very much,” and 4 represented a neutral response. This scale enabled us to capture the extent to which each ideation method was perceived to support creativity-related experiences. By quantifying these subjective perceptions, we were able to compare the effectiveness of traditional (unaided and Internet-assisted) and GenAI-assisted ideation methods in supporting various aspects of the creative process.

## 4 Results

A total of 41 datasets were collected and analyzed using SPSS version 25, with no extreme values detected. Reliability analysis yielded a Cronbach's alpha coefficient of 0.816, which surpasses the conventional threshold of 0.8, thereby indicating satisfactory internal consistency (Cortina, 1993). Inter-rater reliability among the three expert raters was assessed using a two-way random-effects intraclass correlation coefficient (ICC). The ICC values for the average measures of creativity across each metric ranged from 0.86 to 0.99, demonstrating good or excellent reliability and strong consistency across raters. Prior to conducting the repeated-measures ANOVA, the assumption of sphericity was tested using Mauchly's test for each dependent variable. When the sphericity assumption was violated (*p* < 0.05), the Greenhouse-Geisser correction was applied to adjust the degrees of freedom. Partial eta-squared (η_p_^2^) was used as the measure of effect size, with values interpreted as small (0.01), medium (0.06), and large (0.14) according to Cohen's (1988) guidelines. For all significant main effects, *post hoc* pairwise comparisons were performed using the Bonferroni correction to control for Type I error inflation.

**Figure 3:**
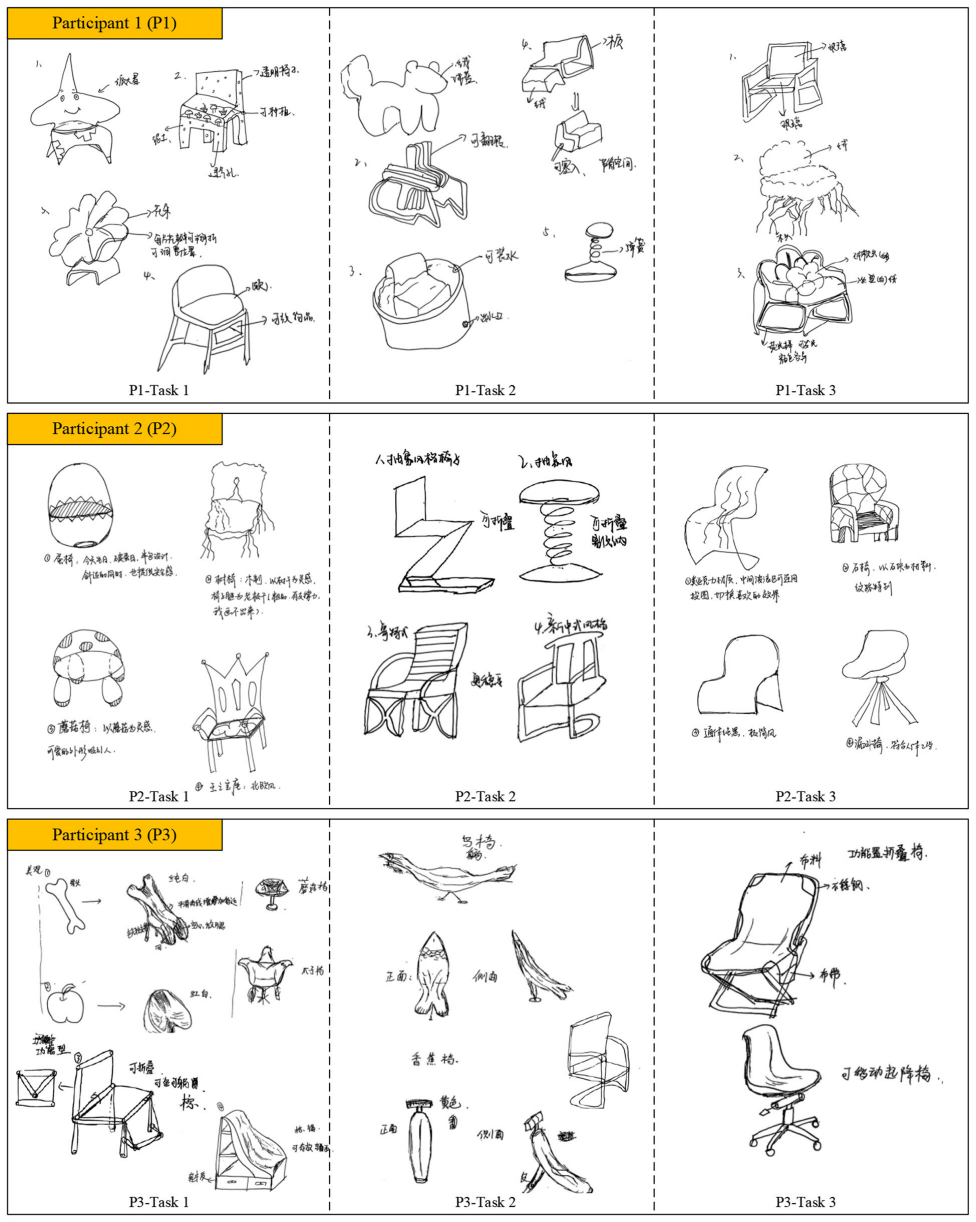
Examples of chair design sketches by participants in each experimental condition.

Subsequently, a 3 × 2 repeated-measures ANOVA was conducted to analyze the variables. Table 2 presents the means and standard deviations of creativity scores for each ideation method, while Figure 3 provides selected examples of participants' design outcomes.

**Table 2:** Mean values (SDs) of the creativity scores for each ideation method regarding the design outcomes and perceived creativity support.

Creativity measures	Creativity metrics	Ideation methods					
		Unaided		Internet-assisted		GenAI-assisted	
		Average	Good	Average	Good	Average	Good
Design Outcomes	Fluency	2.53 (1.02)	3.45 (2.09)	3.02 (1.72)	2.79 (1.40)	1.89 (0.81)	2.45 (1.34)
	Flexibility	3.35 (0.77)	3.52 (0.88)	3.30 (0.62)	3.65 (0.77)	2.77 (0.73)	3.08(0.68)
	Originality	3.18 (0.87)	3.29 (0.67)	3.32 (0.81)	3.64 (0.58)	2.89 (0.99)	2.98 (0.60)
	Elaboration	3.44 (0.83)	3.44 (0.69)	3.56 (0.74)	3.59 (0.89)	3.37 (1.23)	3.58 (1.00)
Perceived Creativity Support	Usability	5.05 (1.22)	4.95 (1.21)	5.26 (1.10)	5.55 (0.80)	5.47 (1.22)	5.86 (0.83)
	Exploration	4.63 (1.21)	4.82 (1.40)	5.53 (0.90)	5.45 (0.91)	5.53 (0.84)	5.50 (0.91)
	Expressiveness	4.95 (1.22)	5.09 (1.23)	5.16 (1.01)	5.50 (0.80)	5.32 (1.00)	5.45 (1.26)
	Immersion	4.63 (1.01)	4.73 (1.28)	5.68 (0.75)	5.14 (1.28)	5.42 (0.84)	5.64 (1.09)
	Enjoyment	4.84 (1.12)	4.82 (1.37)	5.16 (1.07)	5.18 (0.96)	5.37 (1.07)	5.27 (1.28)
	Result worth effort	5.37 (1.01)	5.05 (1.25)	5.53 (0.51)	5.59 (0.85)	5.32 (0.89)	5.27 (1.24)

### 4.1 Design outcome quality

Mauchly's test indicated that the assumption of sphericity was met for fluency, flexibility, and originality (*p* > 0.05), but was violated for elaboration (*p* < 0.001). Accordingly, Greenhouse–Geisser-corrected degrees of freedom were applied for the analysis of elaboration.

When comparing the creativity of design outcomes of three ideation methods during the divergent thinking phase, significant main effects were found across several creativity metrics: fluency [*F*(2,38) = 5.583, *p* = 0.002, η_p_^2^ = 0.289], flexibility [*F*(2,38) = 12.724, *p* < 0.001, η_p_^2^ = 0.401], and originality [*F*(2,38) = 10.170, *p* < 0.001, η_p_^2^ = 0.349]. According to Cohen's (1988) guidelines, all three effect sizes were large.

**Figure 4:**
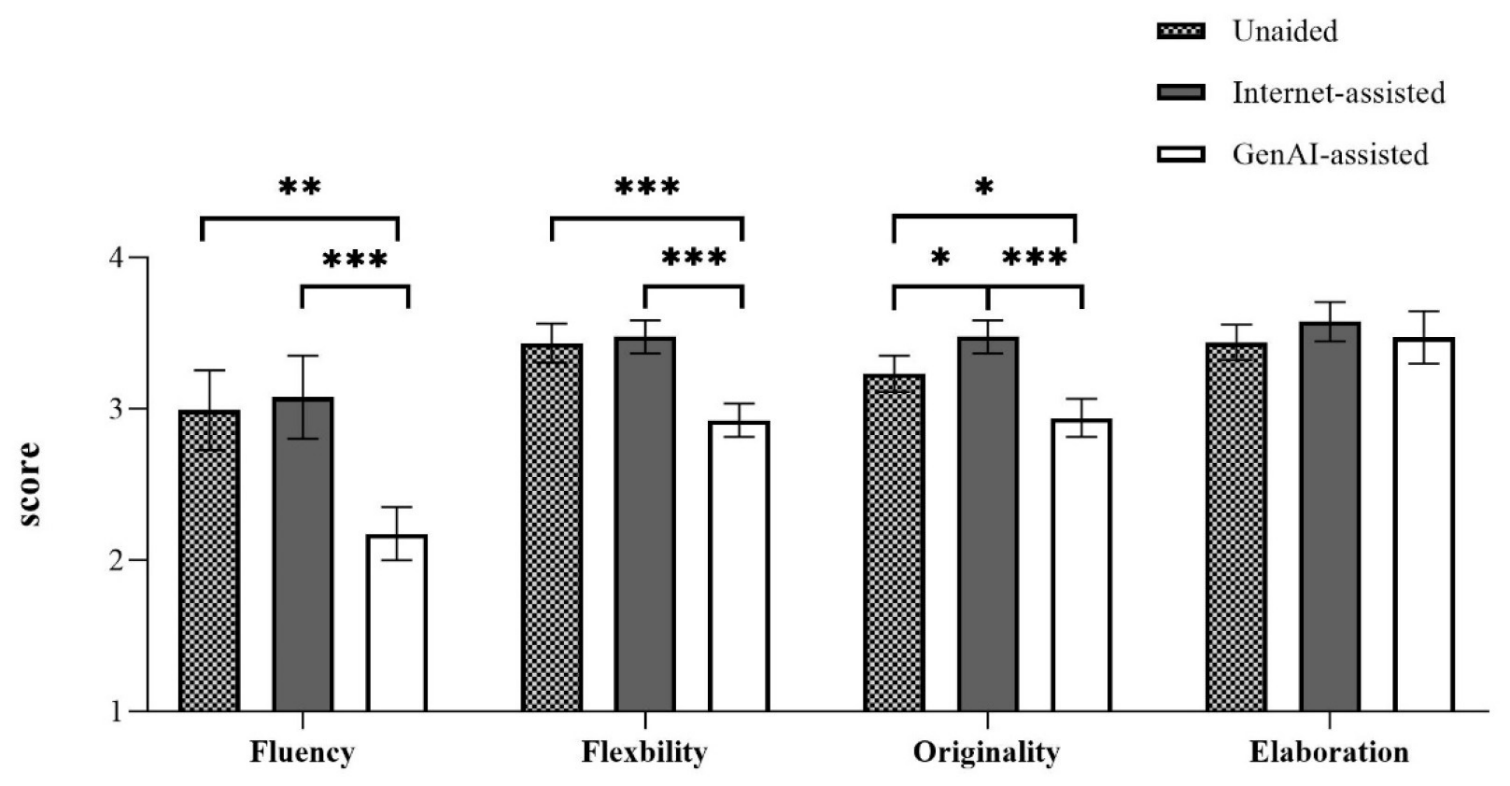
The average creativity scores and standard errors for the three ideation methods. *** statistically significant < 0.001 level, ** statistically significant < 0.01 level, * statistically significant at 0.05 level.

Bonferroni-corrected pairwise comparisons revealed that, for both fluency and flexibility, the Internet-assisted method significantly outperformed the GenAI-assisted method (both ps < 0.001). Similarly, the unaided method produced significantly higher fluency (*p* = 0.002) and flexibility (*p* < 0.001) scores than the GenAI-assisted method. For originality, the Internet-assisted method yielded significantly higher scores than both the unaided method (*p* = 0.036) and the GenAI-assisted method (*p* < 0.001; see Figure 4).

No significant main effect of ideation method was found for elaboration (*p* > 0.05). Furthermore, neither the main effect of creative tendency nor the interaction between ideation method and creative tendency reached statistical significance for any of the design outcome measures (all ps > 0.05).

### 4.2 Perceived creativity support

Mauchly's test indicated that the assumption of sphericity was satisfied for enjoyment (*p* = 0.579) but violated for usability (*p* = 0.004), exploration (*p* = 0.002), expressiveness (*p* = 0.002), immersion (*p* < 0.001), and result worth effort (*p* < 0.001). Greenhouse–Geisser corrections were therefore applied to these latter measures.

In comparing the three ideation methods in terms of perceived creativity support, significant main effects of the design method were observed across several metrics: usability [*F*(2,38) = 10.049, *p* < 0.001, η_p_^2^ = 0.346], exploration [*F*(2,38) = 7.983, *p* = 0.001, η_p_^2^ = 0.296], immersion [*F*(2,38) = 6.459, *p* = 0.004, η_p_^2^ = 0.254], and result worth effort [*F*(2,38) = 3.955, *p* = 0.028, η_p_^2^ = 0.172]. According to Cohen's (1988) guidelines, all effect sizes were large.

**Figure 5:**
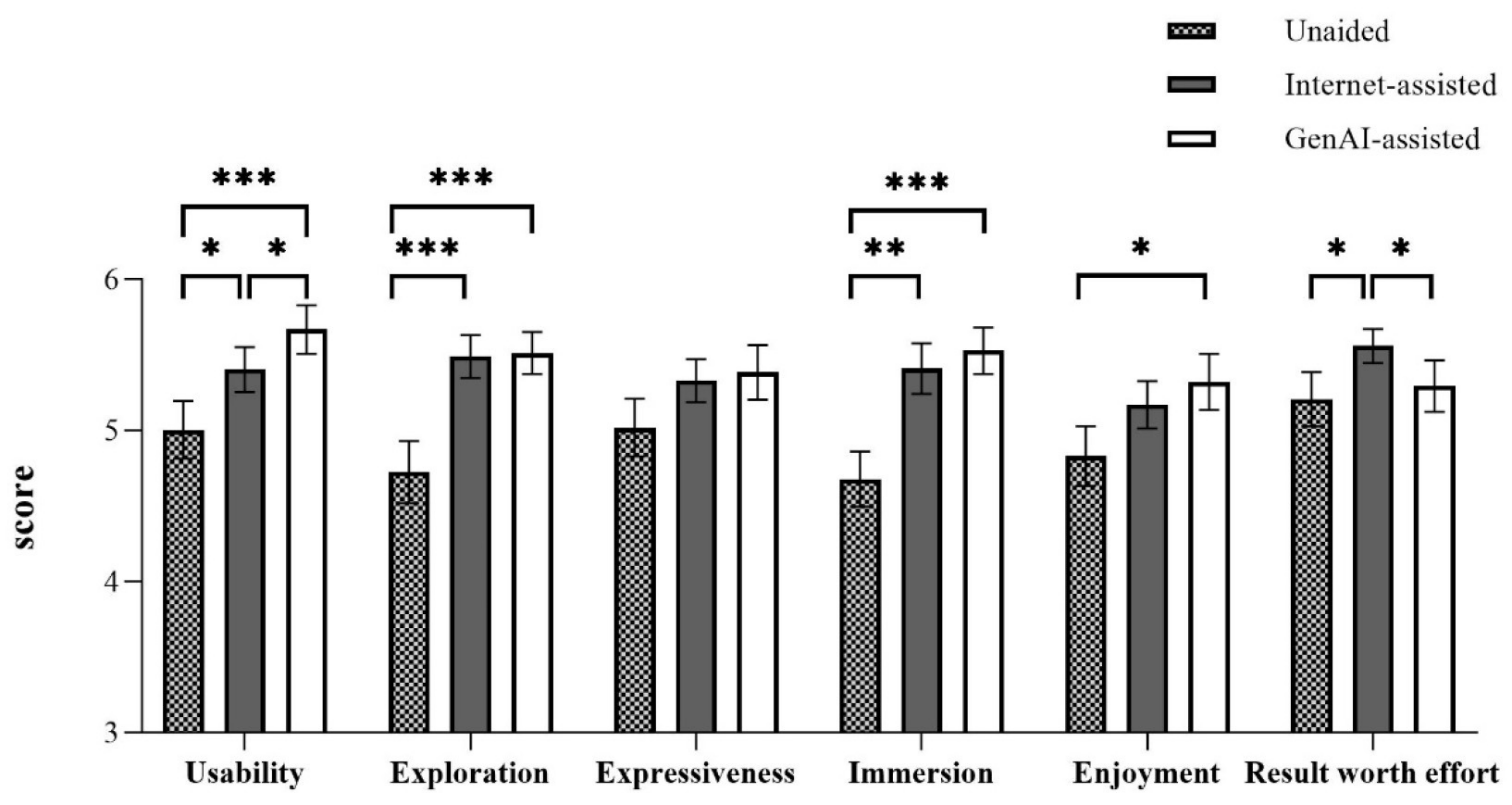
The average perceived creativity scores and standard errors for the three ideation methods. *** statistically significant < 0.001 level, ** statistically significant < 0.01 level, * statistically significant at 0.05 level.

No significant main effects were found for expressiveness or enjoyment. In addition, creative tendency did not significantly affect perceived creativity support across any of the ideation methods (see Figure 5).

Bonferroni-corrected pairwise comparisons revealed that, for usability, the GenAI-assisted method received significantly higher ratings than both the Internet-assisted method (*p* = 0.023) and the unaided method (*p* < 0.001). For exploration, both the GenAI-assisted method (*p* < 0.001) and the Internet-assisted method (*p* = 0.004) received significantly higher ratings than the unaided method, whereas no significant difference was observed between the GenAI-assisted and Internet-assisted methods (*p* > 0.05).

For immersion, the GenAI-assisted method was rated significantly higher than the unaided method (*p* < 0.001), while the Internet-assisted method did not differ significantly from either of the other two methods (all ps > 0.05). For result worth effort, the Internet-assisted method received significantly higher ratings than both the unaided method (*p* = 0.037) and the GenAI-assisted method (*p* = 0.040).

No significant pairwise differences were observed for expressiveness (all ps > 0.05). Although one pairwise comparison for enjoyment reached significance (GenAI-assisted vs. unaided, *p* = 0.037), the overall main effect was not significant and should therefore be interpreted with caution.

Beyond assessing participants' design outcomes and subjective evaluations of creativity support, brief semi-structured interviews were conducted to obtain additional qualitative insights into participants' experiences with the three ideation methods. Most participants expressed a preference for unaided ideation (27 out of 41), followed by Internet-based image searches for inspiration (8 out of 41), and GenAI-assisted approaches using text-to-image tools such as Doubao (6 out of 41). Many participants emphasized that independent ideation allowed them to more directly integrate their personal experiences with chairs into the design process. For instance, they could draw on their family experiences and consider factors such as comfort, ease of cleaning, and spatial adaptability. Table 3 summarizes key participant comments on the three ideation methods gathered during the semi-structured interviews.

**Table 3:** Key participant comments on the three ideation methods.

Ideation methods	Proportion of participants who preferred this method	Key comments on the method
Unaided	65.90%	- Enables direct integration of personal experiences with chair design into the creative process - Facilitates deeper reflection on design concepts
Internet-assisted	19.50%	- Allows better control over the scope and type of information retrieved - External stimuli obtained through searches can inspire creativity
GenAI-assisted	14.60%	- Primarily focuses on the visual form of the product - Encourages adjustments to align with GenAI outputs, reducing designers' sense of autonomy and originality - Rapidly generates multiple design variations from simple keywords

When comparing GenAI-assisted and traditional ideation methods, many participants noted that GenAI-generated concepts tended to emphasize the visual appearance of chairs while providing relatively limited support for practical design considerations, such as armrest angles, seat height, and ergonomic functionality. Several participants perceived the generated outputs as relatively standardized, making it more difficult to derive deeper design insights grounded in specific user needs and usage contexts. Some participants further reported that they occasionally adapted their ideas to align with AI-generated suggestions, which they perceived as reducing their control over the design direction and limiting the originality of their concepts. Participants also indicated that highly contextualized design requirements were not always easily captured through either Internet-based searches or GenAI-generated outputs.

By contrast, the GenAI-assisted method was recognized for its ability to rapidly generate multiple design variations from simple keywords, thereby improving efficiency in the early ideation stage. Moreover, when participants encountered creative blocks or struggled to produce initial ideas, the powerful generative capabilities of GenAI tools served as external stimuli that enhanced idea fluency and provided a rich visual reference space to reignite their creative thinking.

Overall, the interview findings suggest that participants perceived a trade-off between the efficiency and inspirational benefits offered by GenAI tools and the greater sense of autonomy and contextual understanding afforded by traditional ideation methods.

## 5 Discussion

In this study, we explored the impact of traditional and GenAI-assisted ideation methods on both cognitive creativity and perceived creativity support during the divergent thinking phase of product design. Drawing on the quantitative and qualitative findings, we addressed three research questions and identified several insights that contribute to a deeper understanding of creativity support in product design contexts.

### 5.1 How do traditional and GenAI-assisted ideation methods affect the creativity of design outcomes during divergent thinking? (RQ1)

Regarding the RQ1, we found that the GenAI-assisted group did not outperform the traditional ideation methods. Instead, traditional methods, particularly the Internet-assisted method, followed by the unaided method, led to higher performance in overall creative task outcomes. Specifically, our findings indicate that traditional ideation methods more effectively supported fluency, flexibility, and originality in the design outcomes. This result is consistent with the findings of Wadinambiarachchi et al. (2024), who showed that AI-generated images increased design fixation and reduced fluency, variety, and originality compared to the baseline condition (no inspiration support). This finding is also consistent with Bieser (2022), who argued that while GenAI tools have the potential to inspire new ideas, they cannot fully replace the originality derived from human observation and deep cognitive processing.

One possible explanation for the superior flexibility and originality observed in the Internet-assisted condition lies in the different cognitive demands imposed by the two ideation methods. Rather than receiving ready-made solutions, participants needed to formulate search terms, evaluate the relevance of retrieved information, compare alternative references, and selectively integrate useful elements into their own designs. This process of searching, filtering, and synthesizing information may stimulate deeper reflection and encourage the formation of novel associations. In contrast, GenAI tends to provide highly synthesized visual outputs immediately, reducing the need for extensive exploration and information processing. While such efficiency can enhance usability and immersion, it may also shorten the cognitive pathways through which original ideas emerge.

The qualitative interview findings further suggest that some participants relied heavily on AI-generated outputs during ideation, focusing primarily on the generated images rather than actively developing alternative concepts. This may not only result in design fixation (Wadinambiarachchi et al., 2024) but also fosters a form of creative laziness, in which individual ideation effort is diminished (Kalving et al., 2024). Koivisto and Grassini (2023) emphasized that although GenAI can excel in certain tasks, the uniqueness and complexity of human creativity remain difficult to replicate or surpass. Furthermore, human–GenAI collaboration itself may introduce additional coordination costs and interactional disruptions, which could negatively affect creative fluency (Simkute et al., 2025; Tankelevitch et al., 2024).

Taken together, these findings suggest that, within the specific context of the present study involving novice product design students, a chair-design task, and relatively short ideation sessions, traditional ideation methods were associated with higher levels of cognitive creativity in terms of fluency, flexibility, and originality. Whether these findings generalize to professional designers, different design domains, or longer ideation periods remains an important question for future research.

In terms of elaboration, no significant differences were found between the traditional and GenAI-assisted ideation methods. Previous studies have reported mixed findings regarding this dimension. For example, Bangerl et al. (2025) reported that participants in the AI group elaborated their ideas significantly less than those in the no-AI group in an AUT test. In contrast, Kim et al. (2016) found that students in a Korean high-tech high school English class using tablet-assisted visual thinking outperformed those receiving traditional instruction in elaboration on creativity tests. These contextual differences may largely account for the divergent findings. The study by Bangerl et al. (2025), which involved mostly male students from a Data Science and AI program at an Austrian university and employed the AUT test, differs from the present research in terms of task type, participant background, and cultural context. Similarly, the context of Kim et al. (2016), a Korean high school English class, also differs substantially from this study. Based on the above, we infer that within the domain of product design, multimodal image stimuli generated by GenAI provide designers with abundant visual resources that facilitate the refinement of design solutions and the deepening of explorations into aesthetics and function. Consequently, no difference in elaboration levels was observed in the GenAI group compared with the traditional method.

Beyond the immediate effects observed in this experiment, prolonged reliance on GenAI may also introduce longer-term risks to creative development. When designers repeatedly depend on AI-generated suggestions as starting points, they may engage less frequently in independent problem framing, idea generation, and critical exploration. Over time, this could weaken the development of divergent thinking skills and increase the likelihood of convergent, homogenized design outcomes, particularly when many users rely on similar models trained on overlapping datasets. Although these long-term effects were beyond the scope of the present study, they deserve further empirical investigation in both educational and professional design contexts.

### 5.2 How do traditional and GenAI-assisted ideation methods influence designers' perceived creativity support? (RQ2)

Regarding the RQ2, our findings reveal that the perceived creative support provided by different ideation methods shows both significant and nuanced differences. Designers' perceptions are not limited to the functional effectiveness of a tool, but also reflect how these tools interact with their cognitive and affective processes, ultimately shaping the overall creative experience.

In terms of usability, GenAI-assisted methods scored significantly higher than both Internet-assisted and unaided ideation approaches. This finding highlights a core strength of GenAI as a creativity support tool, as it lowers the threshold for idea generation and streamlines the human-AI co-creation process. As a result, designers can focus more directly on conceptual exploration rather than on information retrieval or search strategies. One possible explanation is that GenAI systems provide natural-language interfaces that align closely with everyday communication practices. Rather than relying on specialized keyword strategies or complex search procedures, users can generate content through relatively simple descriptive prompts. This may reduce the cognitive effort required to initiate idea generation (Chandrasekera et al., 2025; Redifer et al., 2019, 2021). Such an intuitive natural-language interaction model diminishes the skill barriers associated with traditional digital tools and makes creative resources more accessible, even for novice designers or participants without professional backgrounds (Shneiderman, 2007).

In terms of exploration and immersion, ideation methods supported by GenAI achieved the highest ratings, followed by Internet-assisted methods, both of which significantly outperformed unaided ideation. This implies that integrating external resources, whether *via* GenAI or traditional internet searches, enhances designers' ability to generate more diverse and novel ideas in early design stages, while also improving focus and sustained engagement. This finding is consistent with previous studies in design cognition research, which highlight that external stimuli can activate associative thinking and help overcome cognitive fixation (Du et al., 2015; Goldschmidt and Smolkov, 2006). The multimodal output of GenAI or Internet searches, such as text and images, allows abstract or vague mental images to be quickly transformed into clear visual or textual representations, speeding up the shift from internal ideation to external expression (Peschl, 2024). This immediate feedback loop not only makes creative ideation easier but also supports the iterative refinement of ideas (Rahman et al., 2025). Chiou et al. (2023) illustrated that the collaborative use of human insight and GenAI in design not only leads to original modes of self-expression and communication but also injects a fresh perspective into the creative process. Their research highlights how GenAI can serve as a catalyst for unlocking new artistic directions, ultimately demonstrating its ability to complement and elevate human creativity.

In terms of perceived worth effort, the Internet-assisted method was rated significantly higher than both the GenAI-assisted method and the unaided method. This suggests that designers considered the time and mental effort spent on Internet searches more valuable. They perceived Internet-assisted approaches as more reliable, offering greater control and richer contextual details. This experience of controlled exploration aligns with Pirolli and Card's (1995) information foraging theory, which explains how people follow “information scents” to locate valuable resources. The Internet, as the core of the information superhighway, operates through mechanisms that reinforce the perception that the effort invested in online searches is well rewarded.

The widespread availability of the Internet also provides tremendous opportunities for individuals and organizations to leverage its features and services for electronic brainstorming (Siau, 1999). Moreover, the Internet environment is particularly conducive to enhancing designers' creativity because of the diverse modalities it offers, such as images, sound, videos, animations, and text (Shoshani and Braun Hazi, 2007). Internet searches further strengthened designers' sense of control and predictability. By selecting specific keywords, they could direct the search process and anticipate both the types of sources, including design platforms and professional databases, and the results they might encounter.

In contrast, although GenAI tools were praised for their speed and potential as a catalyst for creativity, they also risk producing conventional outcomes due to biases in training data (Holzner et al., 2025). Bender et al. (2021) noted that outputs from large language models are generated from patterns learned from existing data, which may constrain the production of highly novel ideas. Considerable time should therefore be devoted to assembling task-specific datasets rather than relying on vast quantities of conveniently scraped online data. Furthermore, the “black-box” nature of GenAI means that generated images can be unpredictable and vary in quality, requiring designers to expend additional effort refining prompts or filtering outputs. This extra workload diminishes GenAI's efficiency advantage and reduces the perceived value of the effort invested.

For expressiveness, no significant differences were observed among the three ideation methods, indicating that participants perceived similar levels of support for articulating and externalizing their ideas. This finding suggests that all three approaches were capable of supporting creative expression, albeit through different mechanisms.

For enjoyment, the overall main effect was not significant, although the GenAI-assisted method received significantly higher ratings than the unaided method in one pairwise comparison. Given the absence of a significant overall effect, these findings should be interpreted cautiously. Nevertheless, the result may indicate that GenAI introduces elements of novelty and surprise that some participants find engaging. Previous research has reported differences in creative outcomes between GenAI-assisted and traditional ideation approaches without necessarily identifying corresponding differences in user satisfaction or engagement (Baltà-Salvador et al., 2025). In this study, each ideation method appears to satisfy distinct psychological needs: unaided ideation fosters a sense of autonomy, Internet searches reinforce competence through structured information access, and GenAI introduces elements of novelty and surprise. This interpretation aligns with research suggesting that perceived enjoyment plays a central role in the adoption of AI tools, even when objective performance outcomes vary (Cano and Nunez, 2024). Given the limited number of studies directly comparing these ideation approaches, further research is needed to better understand how affective experiences such as enjoyment, immersion, and expressiveness evolve throughout the creative process. Future studies employing more fine-grained methods, such as real-time affective tracking or physiological measures, may provide deeper insights into these dynamics.

### 5.3 Do designers with different levels of creative tendency respond differently to traditional and GenAI-assisted ideation methods? (RQ3)

Regarding the RQ3, the findings revealed no significant differences between participants with average and good levels of creative tendency across the three ideation methods. Nevertheless, participants with higher creative tendency scores generally obtained slightly higher creativity scores in their chair design proposals. Although this pattern was not statistically significant, it suggests a possible trend that warrants further investigation. Under the present experimental conditions, individuals with average and good levels of creative tendency appeared to receive comparable support from the three ideation approaches.

However, this non-significant finding should be interpreted cautiously. The distribution of creative tendency scores was relatively restricted, as no participants fell within either the highest or lowest creativity categories defined by the assessment instrument. This limited variability may have reduced the likelihood of detecting potential moderating effects. Furthermore, the relatively simple chair-design task and the modest sample size (*n* = 41) may have resulted in insufficient statistical power to identify small-to-medium interaction effects. Accordingly, creative tendency cannot be dismissed as irrelevant. Its moderating influence may be context-dependent, or it may only become evident when score variability is greater.

This result aligns with previous research indicating that, although personality traits and behavioral indicators are strongly associated with originality and highly creative outcomes, they are not, by themselves, sufficient to ensure such results (Rosar and Weidlich, 2022; Torrance, 2017). Choi (2004) further demonstrated that creative tendency and motivation influence creative performance through creative ability, which is itself a product of divergent thinking. As a motivational personality trait (Eysenck, 1993), creative tendency may exert its influence on design outcomes through various mediating variables such as domain knowledge (DiStefano et al., 2025), task complexity (Cheng and Zhang, 2025), and stimuli features (Yuan et al., 2022).

Previous research has also suggested that external support tools may reduce performance differences associated with individual creative characteristics. For example, Doshi and Hauser (2024) found that less creative writers benefited substantially from access to GenAI-generated ideas, producing stories that were evaluated as more creative and enjoyable. Although no comparable moderating effect was observed in the present study, such findings raise the possibility that external ideation supports, including both GenAI systems and Internet-based resources, may partially compensate for differences in individual creative dispositions under certain conditions. Further research is needed to determine whether such compensatory effects occur in product design contexts.

In the present study, no significant differences were observed between the two creative tendency groups across any of the ideation conditions. One possible explanation is that the chair-design task provided a relatively familiar and constrained design problem, thereby reducing the extent to which individual differences in creative tendency could manifest themselves. More open-ended or complex design challenges may produce different results.

Furthermore, this study focused primarily on the divergent thinking phase of ideation, leaving the convergent phase unexplored. During divergence, creative tendency did not exhibit a significant effect; however, it may exert stronger influences during convergence, when idea evaluation and refinement occur. Future research should therefore examine its role across both divergent and convergent stages to clarify finer distinctions and the underlying cognitive mechanisms.

A further limitation concerns the composition of the sample. Participants' creative tendency scores were concentrated within the average-to-good range, resulting in limited between-group variability. Consequently, potential differences associated with exceptionally high or exceptionally low levels of creative tendency could not be examined. Future studies should recruit participants representing a broader spectrum of creative tendency and employ less structured, more open-ended design tasks. Such research would help clarify whether task characteristics moderate the influence of creative tendency and would further extend the present findings.

### 5.4 Implications

Given that creativity is a complex and multi-faceted construct, this study provides a nuanced perspective on how different ideation methods influence both cognitive creativity and perceived creativity support during product design ideation.

#### 5.4.1 Theoretical implications

This study contributes to the growing body of literature on creativity, design cognition, and human–AI collaboration by providing empirical evidence on how GenAI reshapes divergent thinking in product design. The findings extend theoretical understanding in several important ways.

First, the results provide empirical support for distinguishing between cognitive creativity outcomes and perceived creativity support experiences during the ideation process. While traditional methods outperformed GenAI-assisted ideation on objective measures of fluency, flexibility, and originality, GenAI enhanced designers' subjective perceptions of creative support and engagement. This pattern suggests that creativity may be understood not only as an outcome-oriented construct but also as an experiential process shaped by cognitive, interactional, and affective factors.

Second, the absence of significant moderating effects from creative tendency in this study should not be interpreted as challenging traditional trait-based models. Rather, it highlights the context-dependent nature of trait-expression and the potential for external tools to provide compensatory support that temporarily reduces individual differences in creative output. This aligns with situated cognition perspectives, which emphasize that creativity emerges from person-tool-environment interactions rather than stable internal attributes alone. The finding that GenAI and Internet-assisted methods provided comparable support across average and good tendency levels suggests that, when sufficient external scaffolding is available, the influence of individual creative traits may be reduced. This pattern is also consistent with the compensatory explanation proposed by Doshi and Hauser (2024).

Finally, the findings contribute to emerging discussions of human–AI co-creativity by suggesting that GenAI may function more effectively as an augmentative rather than a substitutive creative partner. Rather than replacing human ideation, GenAI appears to support specific aspects of the creative process, particularly those related to exploration, immersion, and perceived usability. These findings highlight the importance of conceptualizing co-creativity as a dynamic interaction between human agency and computational support.

#### 5.4.2 Practical implications

For design education, the results underscore the potential of GenAI as a pedagogical scaffold that enhances students' perceived creative support and emotional engagement. Integrating GenAI tools such as Doubao, DALL·E and Midjourney into early-stage ideation can help students visualize abstract ideas quickly, overcome expressive barriers, and sustain creative motivation. However, educators should carefully design learning activities that encourage critical reflection, independent thinking, and human-led exploration in order to avoid excessive reliance on AI-generated outputs. A hybrid approach that combines GenAI-generated stimuli with traditional brainstorming and sketching activities may represent a promising direction for future educational practice.

For design practitioners and organizations, the findings suggest that GenAI can serve as a valuable co-creative assistant during early-stage product development by accelerating ideation and facilitating the rapid generation of visual concepts. However, given the potential risks of design fixation and homogenization, organizations should regard GenAI as a tool for augmenting rather than replacing human creativity.

For GenAI tool developers, the findings highlight the importance of developing customized GenAI systems tailored to different design skills and personal traits. Moreover, the study reveals that current “black-box” generative processes often produce unpredictable outcomes, which can make designers feel a lack of control and diminish their perceived value of effort. By drawing inspiration from traditional Internet-based design mechanisms and enhancing transparency, controllability, and feedback functions in GenAI systems, developers can foster greater user trust, engagement, and sense of control, ultimately stimulating designers' creativity.

### 5.5 Limitations and future research

Several limitations of the present study suggest directions for future research.

First, this study primarily focused on the early stage of ideation, that is, divergent thinking, while the later stage of convergent thinking was not examined. As design processes typically encompass both divergent and convergent phases (Yin H. et al., 2023), and designers often shift between these two modes as “a hallmark of creative thinking” (Goldschmidt, 2016), examining how traditional and GenAI-assisted tools influence creativity across the integrated design process would be highly meaningful. Future research should therefore explore the effects of different ideation methods across both stages to develop a more holistic understanding of creative cognition.

Second, the absence of significant moderation effects for creative tendency should be interpreted cautiously. *N*o participants fell into the high or low creativity categories, resulting in a restricted score range that may have limited variability and statistical power to detect group differences. Additionally, the relatively simple chair design task may not have demanded sufficient creative resources to reveal individual differences in creative tendency. Consequently, we cannot conclude that creative tendency is unimportant; rather, its potential moderating role may become apparent in more complex tasks or with greater score variability.

Third, the use of novice product design students from a single Chinese university limits generalizability. Designers with varying expertise levels, disciplinary backgrounds (e.g., architecture, fashion design), or cultural contexts may respond differently to GenAI-assisted ideation. Future studies should therefore test these findings across diverse populations and design domains beyond chair design, such as consumer electronics or household goods. More complex design problems, such as smart products, medical devices, or multifunctional systems, may require broader knowledge integration and deeper problem-solving processes. Under such conditions, the relative advantages of Internet-assisted and GenAI-assisted ideation may differ and warrant further investigation. Additionally, the findings are specific to Doubao, a text-to-image GenAI tool with particular interface characteristics and output unpredictability. Other platforms (e.g., Midjourney, DALL·E, Stable Diffusion) differ in controllability, generation speed, and visual style, which may alter designers' creative responses. Cross-platform comparative studies are needed to clarify whether these patterns generalize across GenAI systems.

Fourth, the three ideation conditions differed inherently in cognitive workflow: unaided ideation relied on internal retrieval, Internet-assisted ideation on external search, and GenAI-assisted ideation on prompt engineering and iterative refinement. These additional interaction demands in the GenAI condition, particularly output unpredictability and tool initialization time, may have influenced performance independently of creativity. Although standardized prompt training was provided to standardize baseline proficiency, the 10-min timeframe may have disproportionately disadvantaged the GenAI condition. Future studies should employ curated image databases as more comparable controls, match conditions on productive ideation time rather than total session time, and statistically control for AI interaction proficiency.

Fifth, the analyses relied on conventional repeated-measures ANOVA, which assumes sphericity and treats participant effects as fixed. While this approach is standard in experimental creativity research and appropriate for our balanced design, linear mixed-effects models (LMMs) would offer more flexible handling of participant-level random effects (Baayen et al., 2008). Future research with larger, more heterogeneous samples should consider LMMs to better account for nested data structures.

Finally, creativity stimulation or inhibition is shaped by multiple contextual variables, including designers' backgrounds, design contexts, task characteristics, and the specific tools employed. These factors may jointly determine how traditional and GenAI-assisted ideation methods influence creative performance. Consequently, future research should adopt a more nuanced and comparative approach to identify both universal and context-specific mechanisms underlying traditional and GenAI-assisted creativity.

## 6 Conclusion

This study investigated the effects of traditional and GenAI-assisted ideation methods on designers' creativity during the divergent thinking stage of product design, focusing on both the creativity of design outcomes and designers' self-reported user experiences. The results revealed that, among novice product design students, traditional ideation methods yielded higher levels of fluency, flexibility, and originality than GenAI-assisted methods. In contrast, GenAI tools were perceived as highly usable and effective in supporting idea exploration, engagement, and immersion during the ideation process. Furthermore, no significant moderating effect of creative tendency was observed across the three ideation methods.

These findings contribute to a deeper understanding of how GenAI influences creative ideation among novice designers in controlled educational settings. Extending these findings to professional design practice requires further empirical validation. Although GenAI tools did not outperform traditional methods in generating highly original design outcomes, they demonstrated clear value in enhancing designers' perceived creative support and engagement. Integrating both approaches in design education and professional contexts can promote richer and more effective creative processes by combining the strengths of traditional and GenAI-assisted ideation. Furthermore, the study underscores the potential of human–AI collaboration to enhance creativity by supporting cognitive processes, expanding the creative space, and accommodating individual differences. It points toward a future where AI serves as a complementary partner in the design process rather than a replacement for human ingenuity.

## Data Availability

The raw data supporting the conclusions of this article will be made available by the authors, without undue reservation.
